# Comparative Evaluation of Analgesic Efficacy of Ultrasound-Guided Pericapsular Nerve Group Block and Femoral Nerve Block During Positioning of Patients With Hip Fractures for Spinal Anesthesia: A Prospective, Double-Blind, Randomized Controlled Study

**DOI:** 10.7759/cureus.56270

**Published:** 2024-03-16

**Authors:** Annamale Jeevendiran, Srinivasan Suganya, Chinthavali Sujatha, Jayashridevi Rajaraman, Surya R, Arthi Asokan, Radhakrishnan A

**Affiliations:** 1 Anaesthesiology and Critical Care, Sri Venkateshwaraa Medical College Hospital and Research Centre, Puducherry, IND; 2 Anaesthesiology and Critical Care, Saveetha Medical College and Hospital, Chennai, IND

**Keywords:** ultrasound-guided nerve block, perioperative analgesia, femoral nerve block, hip fractures, positional pain, spinal anesthesia, peng block

## Abstract

Introduction: Hip fractures cause severe pain during positioning for spinal anesthesia (SA). Intravenous systemic analgesics can lead to various complications in elderly patients, hence peripheral nerve blocks are emerging as a standard of care in pain management for hip fractures, among which femoral nerve block (FNB) is widely known and practiced. Pericapsular nerve group (PENG) block is a recently described technique that blocks the articular nerves of the hip with motor-sparing effects and is used to manage positional pain in hip fractures. This study aims to evaluate the analgesic efficacy of PENG block over FNB in managing pain during positioning before SA in hip fractures.

Materials and methods: This was a prospective, randomized, double-blinded study. After ethical clearance, 70 patients undergoing hip fracture surgery under SA in a tertiary-care hospital were recruited and randomized to receive either ultrasound-guided PENG block or FNB with 20 ml of 0.25% bupivacaine before performing SA. We compared pain severity using the visual analog scale (VAS) 15 and 30 minutes after the block and during positioning. The sitting angle, requirement of rescue analgesia for positioning, and anesthesiologist and patient satisfaction scores were also analyzed. Continuous data were analyzed with an unpaired t-test while the chi-square test was used for categorical data.

Results: There was a significant reduction in VAS scores after PENG block (PENG: 0.66 ± 1.05 and FNB: 1.94 ± 1.90; p = 0.001) with lesser requirement of rescue analgesia for positioning compared to FNB. The anesthesiologist and patient satisfaction scores were also significantly better in the PENG group.

Conclusion: PENG block offers better analgesia for positioning before SA than FNB without any significant side effects, and improves patient and anesthesiologist satisfaction, thus proving to be an effective analgesic alternative for painful hip fractures.

## Introduction

Regional anesthesia is widely preferred for hip surgeries over general anesthesia in elderly individuals, owing to its distinctive advantages, such as reduced length of hospital stay and lesser incidence of blood loss, thromboembolism, pneumonia, morbidity, and mortality [1-3]. Patients with hip fractures suffer from severe pain, which makes it challenging to achieve optimal positioning for spinal anesthesia (SA) [4]. Alleviating positional pain is necessary as it increases patient comfort and provides optimal positioning for administering SA [5]. Systemic analgesics and sedatives that are commonly used to treat acute and positional pain in hip fractures include opioids, non-steroidal anti-inflammatory drugs (NSAIDs), ketamine, dexmedetomidine, etc., and each group of drugs is known to cause specific adverse effects, especially in the elderly population, like respiratory depression, urinary retention, sedation, cognitive impairment, delirium, acute kidney injury, emergence reactions, and bradycardia [2,3,6]. Ultrasound-guided peripheral nerve blocks have become the standard of care in treating acute pain, procedural pain, and as a part of anesthetic management for hip fractures, as they provide superior analgesia with minimal adverse effects [7-10].

Various peripheral nerve blocks, such as femoral nerve block (FNB), fascia iliaca block (FIB), and lumbar plexus block, have been described in the literature as analgesic options for hip fractures but each has its limitations [11-14]. Girón-Arango et al. described the novel pericapsular nerve group (PENG) block, which targets the articular branches supplying the anterior capsule of the hip joint as they cross the anterior inferior iliac spine and iliopubic eminence, providing effective analgesia without affecting motor function [15,16]. FNB is one of the existing standard practices for pain management in hip fractures and there are very few research studies comparing the efficacy of the two blocks for positional pain. This study aimed to compare the analgesic efficacy of ultrasound-guided PENG block and FNB for positional pain in hip fracture patients before administering SA. We hypothesized that the PENG block would offer better analgesia over FNB.

## Materials and methods

After obtaining the Institutional Ethical Committee approval (SVMCH/IEC/2021-FEB/IEC-51) and registering with the Clinical Trials Registry of India (CTRI/2022/08/044619), this prospective, double-blinded, randomized controlled trial was initiated. The study was conducted between August 2022 and January 2023, where we recruited 70 individuals with painful hip fractures (neck of femur, intertrochanteric, and subtrochanteric fractures), belonging to the American Society of Anesthesiologists - Physical Status (ASA-PS) Class I, II, and III, posted for surgery under SA with expected duration of less than three hours, after receiving written informed consent. Patients with polytrauma, morbid obesity, neurocognitive and psychiatric disorders, and any contraindication to regional or neuraxial block like allergy to local anesthetics, hemorrhagic diathesis, infection over the injection site, and spinal deformities were excluded.

The participants were randomized to receive an ultrasound-guided PENG block or FNB using a computer-generated block randomization sequence and allocation was done using a serially numbered opaque sealed envelope approach. The block was performed by an experienced anesthesiologist who was not involved in further study and data collection. Patients and the anesthesiologist collecting the data were blinded to the randomization.

Patients were explained about the procedure and the visual analog scale (VAS) during pre-anesthetic assessment. After shifting the patients to the operating room, standard monitoring like pulse oximeter, noninvasive blood pressure, and an electrocardiogram (ECG) were attached and recorded. Pre-procedure pain using VAS (marked on a continuous 10 cm line denoting 0 - no pain and 10 - worst possible pain) was assessed at rest and with movement (passive leg raise (PLR) to 15°). For PLR, with the patient in the supine position, the affected limb was gently raised to approximately 15° with slight flexion of the hip and knee in extension.

**Figure 1:**
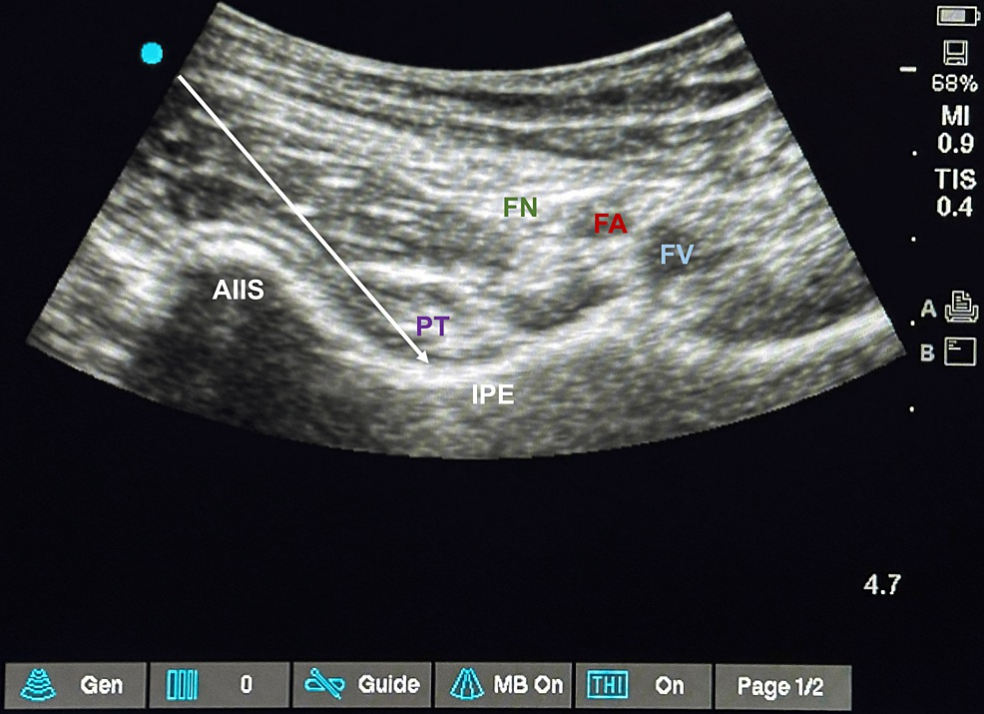
Sonoanatomy of pericapsular nerve group (PENG) block AIIS: anterior inferior iliac spine; PT: psoas tendon; IPE: iliopectineal eminence; FA: femoral artery; FV: femoral vein; FN: femoral nerve; white arrow: needle path and tip at the target site.

For the PENG block, we used a curvilinear, low-frequency (2-5 MHz) ultrasound transducer (Fujifilm Sonosite Edge 2, Bothell, WA), for performing the block. A curvilinear probe was placed in the transverse plane to identify the anterior inferior iliac spine (AIIS) and then rotated about 45° counter-clockwise to align with the pubic ramus visualizing the iliopectineal eminence, Iliopsoas muscle and tendon, femoral artery, and nerve [16]. For this procedure, an 80 mm, 22-gauge echogenic needle was inserted in-plane from lateral to medial, with the tip targeted toward the plane between the psoas tendon and the pubic ramus (Figure 1). After negative aspiration, a local anesthetic (LA) solution (20 ml of 0.25% bupivacaine) was administered in 5 ml increments.

**Figure 2:**
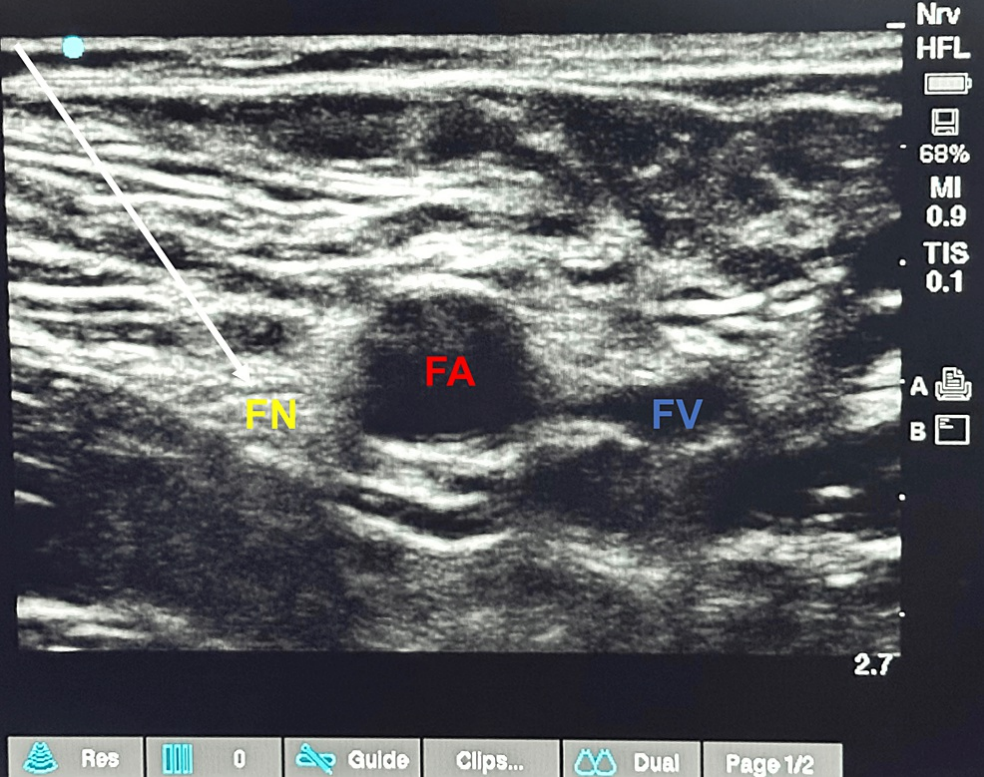
Sonoanatomy of femoral nerve block FN: femoral nerve; FA: femoral artery; FV: femoral vein; white arrow: needle path and tip at the target site.

For FNB, patients were positioned supine, a linear ultrasound probe (4-13 MHz) was used to locate the femoral vessels in the groin skin crease in a transverse plane, and the probe was moved cranially to ensure it was positioned cranially to the bifurcation of the artery. The nerve was identified as an echogenic triangular structure lateral to the femoral artery, and the needle was inserted lateral to medial using an in-plane technique until the tip was positioned immediately adjacent to the femoral nerve (Figure 2). After negative aspiration, 20 ml of 0.25% bupivacaine solution was injected under ultrasound visualization, with 5 ml increments [17].

Patients were continuously monitored for hemodynamic parameters like heart rate, mean arterial pressure, and blood oxygen saturation. They were monitored for any local anesthetic systemic toxicity (LAST) symptoms for 30 minutes and other block-related complications. Pain intensity was assessed during rest and movement with the help of VAS at 15 and 30 minutes after the block. If any patient had a VAS ≥ 3 after 30 minutes of the block, it was considered block failure, and Intravenous (IV) fentanyl 0.5-1 μg/kg was used as analgesia before positioning the patient to sit for SA. The sitting angle was observed subjectively (as leaning forward, sitting straight, or leaning backward), and the anesthesiologist satisfaction score for performing SA (not satisfactory, satisfactory, good, excellent) was also noted [18]. Under aseptic precautions, SA was given in a sitting position in the midline at the L3-L4 level, with 2 ml of 0.5% hyperbaric bupivacaine (10 mg) and 0.5 ml fentanyl (25 µg) using 90 mm, 25-gauge Quincke needle. We did not use any other additional analgesia intraoperatively. Patients were followed up postoperatively for the duration of analgesia by assessing VAS every two hours till the time VAS score was ≥ 3 or the patient demanded the first analgesic, and any adverse effects were also noted. At the end of 24 hours, patients were asked about their experience with the block to assess patient satisfaction scores, and we noted their responses to the question of whether they would opt for the same block in the future if required.

The primary outcome measures were VAS scores at rest and movement after a PLR to 15°, as well as VAS scores at 15 minutes and 30 minutes after the block. The secondary outcome measures were sitting angle during positioning, the anesthesiologist satisfaction score for performing SA, the requirement of rescue analgesia (IV fentanyl) for reduction of VAS ≤ 3 for positioning, the patient’s satisfaction score, duration of analgesia (time from onset of the block to demand of first analgesic postoperatively), and incidence of block-related complications (LAST, nerve or vascular injury, hematoma, site infection).

Statistical analysis

Since there was no study comparing PENG and FNB at the inception of this study, we obtained mean VAS and standard deviation (SD) during positioning after FNB from one study and similarly for PENG block from another study [19,20]. To detect a minimum expected difference in means of VAS of 0.65, with a power of 80%, and a confidence interval of 95%, considering 10% attrition, the sample size was determined to be 70 with 35 in each group, using statistical software OpenEpi version 3.0.

Normally distributed continuous data like VAS scores between the groups and duration of analgesia were expressed as mean and standard deviation and analyzed using unpaired t-test. Categorical data like sitting angle, requirement of rescue analgesia, and anesthesiologist and patient satisfaction scores were expressed as numbers or percentages and analyzed with the chi-square test. P-value ≤ 0.05 was considered statistically significant. All data were analyzed using SPSS version 24 (IBM Corp., Armonk, NY).

## Results

**Figure 3:**
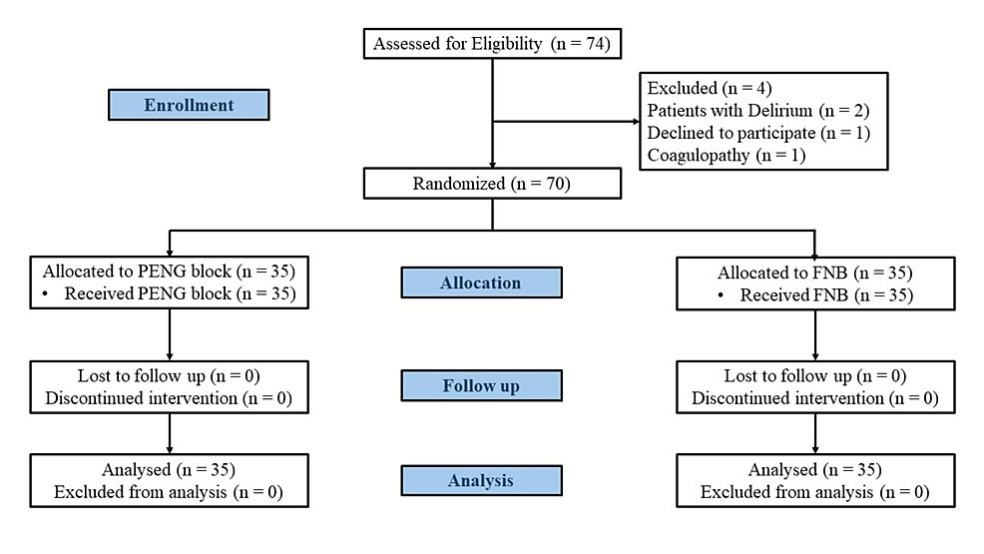
CONSORT diagram CONSORT: Consolidated Standards of Reporting Trials; PENG: pericapsular nerve group block; FNB: femoral nerve block.

A total of 70 patients were recruited and all of them completed the study, and their data were analyzed (Figure 3).

The demographic characteristics, type of fracture, and surgical procedures were comparable (Table 1).

**Table 1:** Comparison of the demographic characteristics, ASA-PS class, and type of fracture between the groups SD: standard deviation; BMI: body mass index; ASA-PS: American Society of Anesthesiologists - Physical Status; NOF: neck of femur; IT: intertrochanteric; ST: subtrochanteric; FNB: femoral nerve block; PENG: pericapsular nerve group.

Variables	PENG (n = 35)	FNB (n = 35)	P-value
Age (mean ± SD, years)	55.69 ± 12.57	53.86 ± 13.69	0.563
Gender			
Male	16	20	0.338
Female	19	15	
BMI (mean ± SD, kg/m^2^)	24.40 ± 2.98	24.85 ± 2.92	0.525
ASA-PS			
I	13	11	0.549
II	15	13	
III	7	11	
Type of fracture			
NOF	18	21	0.763
IT	13	11	
ST	4	3	

The mean VAS scores were comparable in both the groups at rest and movement before the block (p = 0.339 and 0.796, respectively). The mean VAS scores reduced significantly at 15 minutes (PENG: 0.8 ± 1.13 and FNB: 2.43 ± 1.53; p = 0.000) and 30 minutes after block during positioning (PENG: 0.66 ± 1.05 and FNB: 1.94 ± 1.90; p = 0.001) in the PENG group when compared to the FNB group (Table 2).

**Table 2:** Comparing VAS before and after block between the groups * P < 0.05 (statistically significant). VAS: visual analog scale; SD: standard deviation; PLR: passive leg raising; FNB: femoral nerve block; PENG: pericapsular nerve group.

Groups	Pre-block VAS (mean ± SD)		Post-block VAS (mean ± SD)	
	At rest	PLR	Rest (15 min)	Positioning (30 min)
PENG (n = 35)	5.46 ± 0.88	7.36 ± 0.94	0.80 ± 1.13	0.66 ± 1.05
FNB (n = 35)	5.26 ± 0.85	7.69 ± 0.90	2.43 ± 1.53	1.94 ± 1.90
P-value	0.339	0.796	0.000*	0.001*

The sitting angle of the patients was observed to be better and the requirement for supplemental analgesia was significantly lower in the PENG group during positioning (p = 0.001). The anesthesiologist satisfaction score and patient satisfaction score were also significantly better in the PENG group (Table 3).

**Table 3:** Comparison of sitting angle, requirement of rescue analgesia, anesthesiologist and patient satisfaction scores, and duration of analgesia * P < 0.05 (statistically significant). SD: standard deviation; FNB: femoral nerve block; PENG: pericapsular nerve group.

Variables	PENG (n = 35)	FNB (n = 35)	P-value
Sitting angle			
Lean forward	22	9	0.001*
Sitting straight	13	17	
Lean backward	0	9	
Rescue analgesia			
Required	2	9	0.045*
Not required	33	26	
Anesthesiologist satisfaction score			
Not satisfactory	0	2	0.035*
Satisfactory	2	5	
Good	9	15	
Excellent	24	13	
Patient satisfaction score			
Yes	33	27	0.040*
No	2	8	
Duration of analgesia (mean ± SD, hours)	6.66 ± 1.25	5.46 ± 0.85	0.000*
Complications	Nil	Nil	

There was no clinically significant difference in the incidence of hemodynamic changes and other adverse effects observed in both groups (Table 4).

**Table 4:** Hemodynamic parameters before and after the block between the groups * P < 0.05 (statistically significant). SD: standard deviation; HR: heart rate; MAP: mean arterial pressure; SPO2: oxygen saturation; FNB: femoral nerve block; PENG: pericapsular nerve group.

Groups	Pre-block (mean ± SD)			Post-block (mean ± SD)		
	HR	MAP	SPO_2_	HR	MAP	SPO_2_
PENG (n = 35)	86.91 ± 8.51	93.91 ± 4.40	99.29 ± 0.78	78.23 ± 9.45	93.90 ± 4.41	99.43 ± 0.69
FNB (n = 35)	88.94 ± 7.55	92.66 ± 7.78	99.40 ± 0.65	86.54 ± 8.64	92.58 ± 7.63	99.60 ± 0.55
P-value	0.366	0.105	0.147	0.000*	0.378	0.259

## Discussion

SA is the preferred anesthetic technique of choice in orthopedic surgery for lower limb fractures considering its benefits, but patient positioning for SA may cause severe pain and discomfort in patients with hip fractures, thus requiring systemic analgesics to alleviate positional pain. Among the systemic analgesics, opioids are widely used but they are known to be associated with side effects like cognitive impairment, vomiting, urinary retention, and respiratory depression, especially in the elderly [1-5,21]. Hence, many nerve blocks like the three-in-one block, FNB, and fascia iliaca compartment block (FICB) have been tried and tested as an alternative analgesic approach to aid in positioning these patients but their efficacy has been suboptimal, probably owing to their inconsistent blockade of articular branches [16]. FNB has been associated with quadriceps muscle weakness resulting in increased postoperative falls and delayed mobility, warranting an alternative technique devoid of that consequence [12,22,23]. The PENG block is a novel regional analgesic technique described by Girón-Arango et al. for hip fractures, and they observed that all patients had lower pain scores without quadriceps weakness [16]. This is a promising approach as an alternative to existing regional nerve blocks, as it targets only the articular branches from the femoral, obturator, and accessory obturator nerves that supply the anterior capsule of the hip joint as they cross the AIIS and iliopubic eminence, providing effective analgesia without affecting motor function. There are not enough studies comparing PENG with existing regional blocks to incorporate it into routine practice, and almost none with FNB at the time of conception of this study.

The results from this study showed a significant reduction in pain from moderate-severe pain (VAS 5-7) to mild pain (VAS 0-2) at rest and movement, indicating that the PENG block provides better analgesia than FNB for patient positioning in hip fractures (p < 0.05). This pain score reduction was similar to the case series of the original PENG block reported, where they observed a drop of seven points in VAS after the block [16]. Similarly, another case series that performed USG-guided PENG block on nine patients who underwent hip fracture surgeries found a significant reduction in VAS scores at rest (baseline: 6.77 ± 1.20; 30 minutes post block: 0.44 ± 0.52) and with PLR (baseline: 9.22 ± 0.83; 30 minutes post block: 1.44 ± 0.72) [24]. In yet another cohort study performed by Sahoo et al., USG-guided PENG block was administered to 20 consecutive patients posted for hip fracture surgeries and inferred significant drop of six to seven points in VAS scores at rest (baseline: 7.45 ± 1.53; 30 minutes after block: 1.1 ± 1.07) and with movement (baseline: 9.45 ± 0.75; 30 minutes after block: 2.35 ± 1.34) during positioning the patients for SA [25]. There are many other case series, placebo-controlled trials with PENG block, and trials comparing PENG with other established blocks like fascia iliaca block demonstrating similar results [14,18,26-30].

We observed optimal patient positioning, that is, a good sitting angle of leaning forward, 30 minutes after block (63% in PENG vs. 26% in FNB, p = 0.001) and hence a better anesthesiologist satisfaction score for performing SA, which was excellent in 24 patients (68%) in the PENG group compared to 13 patients (37%) in the FNB group, thus suggesting PENG block to offer superior analgesia. These findings were similar to a placebo-controlled study involving 30 patients in each group, demonstrating the effectiveness of PENG block in lowering pain scores during positioning, with a good sitting angle (flexion more than 90°) observed in 87% of the patients who received block compared to only 3% in those who did not receive [18]. Similarly, in a cohort study of 20 patients, they observed satisfaction in terms of ease of positioning patients for SA, which was noted as optimal in 75% of patients [24]. Furthermore, the requirement of rescue analgesia with IV fentanyl during positioning for SA (if VAS ≥ 3) was significantly more in the FNB group (25.7%) when compared to the PENG group (5.7%). This was in concordance with a few randomized trials comparing PENG and fascia iliaca block (FIB) for positioning, where they found that the PENG group offered better patient positioning, and no patients required additional fentanyl for the same [20,27].

Patient satisfaction was observed by their willingness to accept the block in the future, which was better in the PENG group (94%) than in the FNB group (77%). This was similar to other studies that have observed good patient satisfaction scores [20,25,26]. There were no significant hemodynamic changes or block-related adverse effects in both groups.

Studies assessing postoperative pain scores and duration of analgesia have shown significantly lower pain scores (VAS ≤ 4) in the PENG block for four to six hours, lasting for approximately eight to 12 hours with reduced opioid consumption [20,27,29,31]. We also made similar observations where VAS scores were significantly lower in the PENG group than in the FNB group over a period of 24 hours, while duration based on time to first rescue analgesic lasted for 6.6 hours in PENG and 5.5 hours in FNB (p < 0.001). Recently, two studies were published comparing the duration of PENG and FNB in hip fractures. An observational cohort study compared the data of 42 patients who received either PENG or FNB and reported that there was no significant difference in mean postoperative VAS and opioid consumption for 24-48 hours [32]. Another randomized, double-blinded study compared the duration of PENG and FNB based on the ability of patients to recall their sensory and motor recovery, and hence only data from 24 patients (13 in FNB, 11 in PENG) could be analyzed and found, with PENG lasting for 22.5 hours compared to FNB, which lasted for 15.35 hours [33].

Based on our findings in this study, PENG block offered better analgesia than FNB in hip fractures as observed by the significant reduction in VAS scores post-block, resulting in improved patient positioning with reduced opioid consumption and hence improved anesthesiologist and patient satisfaction. We recommend PENG block for perioperative pain management in patients with hip fractures as it provides good analgesia and patient comfort with the least adverse effects, making it a potential alternative to femoral nerve blocks in the future.

This study has some limitations. Firstly, we did not note the cumulative postoperative opioid consumption, which could have been a better predictor of the quality of analgesia. Second, we did not assess the mobility, functional recovery, and length of hospital stay of the patients, which could have given us more data about the potential of PENG block to be a part of the enhanced recovery after surgery (ERAS) for hip fracture repairs. Third, an assessment of motor functions could have been done in the postoperative period to validate the motor-sparing quality described with the PENG block. Further studies are needed to validate the superiority of PENG over existing regional blocks, as it has the potential for better postoperative outcomes with minimal complications.

## Conclusions

PENG block provides better analgesia than FNB in patients with hip fractures during positioning for SA, thereby resulting in improved patient and anesthesiologist satisfaction. Hence, PENG block can be considered an alternative to existing systemic and regional analgesia options for positioning and hip surgeries.
